# Creation of Three-Dimensional Anatomical Vascular and Biliary Models for the Study of the Feline Liver (*Felis silvestris catus* L.): A Comparative CT, Volume Rendering (Vr), Cast and 3D Printing Study

**DOI:** 10.3390/ani13101573

**Published:** 2023-05-09

**Authors:** Daniel Rojo Ríos, Gregorio Ramírez Zarzosa, Marta Soler Laguía, David Kilroy, Francisco Martínez Gomariz, Cayetano Sánchez Collado, Francisco Gil Cano, María I. García García, José Raduán Jáber, Alberto Arencibia Espinosa

**Affiliations:** 1 Department of Anatomy and Comparative Pathological Anatomy, Veterinary Faculty, Campus de Espinardo, University of Murcia, 30100 Murcia, Spain; danielrojo@um.es (D.R.R.); grzar@um.es (G.R.Z.); f.gomariz@colvet.es (F.M.G.); scollado@um.es (C.S.C.); cano@um.es (F.G.C.); 2 Department of Animal Medicine and Surgery, Veterinary Faculty, Campus de Espinardo, University of Murcia, 30100 Murcia, Spain; mtasoler@um.es; 3 Veterinary Science Centre, University College Dublin, Belfield, D04 V1W8 Dublin, Ireland; david.kilroy@ucd.ie; 4 Support Research Service SACE-ACTI, University of Murcia, 30100 Murcia, Spain; mariagarcia@um.es; 5 Department of Morphology, Veterinary Faculty, University of Las Palmas de Gran Canaria, 35413 Las Palmas, Spain; joseraduan.jaber@ulpgc.es

**Keywords:** CTA, liver vascular anatomy, volume rendering, 3D printing

## Abstract

**Simple Summary:**

Vascular liver diseases have been studied in dogs. However, cats are now receiving special attention in veterinary clinics. Therefore, this study focuses on the feline liver with the aim of increasing knowledge on the subjects. Studies on the vascular and biliary system of the feline liver are rare; our literature search found five general studies in manuals, three articles in feline-specific journals, and many others in non-specific studies related to the liver. The feline hepatic parenchyma is traversed by vessels and the biliary system, which can be studied by modern anatomical methods such as vascular and biliary epoxy injections together with diagnostic imaging techniques such as computed tomographic angiography (CTA). The use of three-dimensional (3D) reconstructions (volume rendering) and three-dimensional prints to obtain 3D anatomical prototypes allows for the identification of branches of the arterial, venous and biliary systems and their distribution throughout the liver. We observed that the vascular and biliary structures of the feline liver differ from those of the dog. Finally, we believe that in the future, 3D prototypes could be printed by clinicians to help them in the detection of liver pathology in cats.

**Abstract:**

In this study, six adult feline cadavers were examined using CTA, 3D printing, and casts injected with epoxy. The aorta, the portal vein, and the gallbladder of 3 feline cadavers were separately injected with a 50% mixture of colored vulcanized latex and hydrated barium sulfate as contrast medium to analyze by CT the arterial, venous and biliary systems. The other three cadavers were injected with a mixture of epoxy resin in the aorta, gallbladder and hepatic veins, separately. After the corrosion and washing process, hepatic vascular and biliary casts were obtained. The images obtained by CT showed the vascular and biliary system using a soft tissue window. For the identification of vascular and biliary structures, the 3D prints together with the 3D reconstructions were analyzed, and the results were compared with the casts obtained with epoxy resin. Each of the arterial, venous and biliary branches associated with each of the liver lobes were identified with the help of the printings. In conclusion, the creation of 3D prototypes of nonpathological feline hepatic parenchyma can be used in the veterinary clinic as a basis for the detection of pathological problems in addition to obtaining future pathological hepatic 3D models.

## 1. Introduction

The feline liver is the largest accessory gland associated with the digestive system. Located in the cranial abdomen, it is commonly affected by diseases such as lipidosis, cholangiohepatitis complex, toxic hepatopathy and hepatic neoplasia [1,2,3]. Feline patients are becoming more common in our veterinary clinics; however, most of the studies based on the application of diagnostic imaging techniques have traditionally been carried out in dogs and the results compared with cats [4]. Some scientific studies focused on cats have been conducted, establishing differences between species [1]. Despite this, abdominal cavity studies using diagnostic imaging techniques in cats are still very scarce. Recently, general cat anatomy has been analyzed in [5], which also studied the liver using modern anatomical and diagnosis techniques.

Metzger et al. [6] describe the anatomy of the portal and hepatic veins using corrosion casts and CT of portal and hepatic veins in several healthy cats’ livers. The authors of [7,8] have demonstrated that in cats, rabbits and sheep, significant differences do occur in the intrahepatic distribution of blood.

Gil Cano et al. [9] compared sectional anatomical and CT images of cats, emphasizing the usefulness of anatomical sections in identifying a large number of thoracic and abdominal structures visible in CT images. In 2003, Head et al. [10] attempted to demonstrate the efficacy of computed tomography and radiolabelled granulocytes in the evaluation of the feline pancreas in six disease-free cats and Shojaei et al. [11] analyzed pathological or experimental changes in the feline abdominal region by performing CT studies using iodinated contrast medium.

There are few specific studies on the feline liver using diagnostic imaging techniques. Gaschen [1] highlighted the complementarity of CT and MRI with radiology and ultrasonography to standardize liver studies in healthy and diseased dogs and cats. Cordella and Bertolini [12] described the computed tomographic (CT) features and visualization techniques of hepatic portal venous gas (HPVG) and pneumobilia (PB) in a group of small animal veterinary patients and Larson [2] underlined the use of ultrasound as a valuable noninvasive imaging modality for the evaluation of liver and biliary diseases. Other studies, such as Soler et al. [3], described three different imaging techniques (radiography, ultrasonography and CT) for identifying an accessory liver lobe in dogs and cats [13,14] and only in cats [15], respectively. Lamb [16] carried out a study of the morphology of congenital intrahepatic portosystemic shunts in dogs and cats which can provide a preoperative anatomical assessment that correlates well with the results of portography and may aid in surgical planning. Marolf [17,18] assessed veterinary clinicians and their current use of MRI, CT and advanced ultrasound techniques for the liver, pancreas and biliary system in dogs and cats. Larson [2] evaluated ultrasonography for the normal and abnormal appearance of the liver, gallbladder, bile duct and pancreas of both dogs and cats. Finally, Hespel et al. [19] correlated anatomical slices with CT images to identify vascular and visceral structures and to obtain volumetric reconstructions.

The main objective of this study was to develop an accurate morphological analysis of the feline liver parenchyma through vascular and biliary replications with latex and epoxy and to use computed tomography (CT) with vascular contrast media in cadavers to obtain maximum intensity projection (MIP) images, surface and volumetric reconstructions and, finally, to use 3D printing to produce prototypes.

Works such as Wilhite et al.; Lauridsen et al.; Rojo et al. [20,21,22] show how 3D printing technology provides veterinary medicine with a powerful tool to facilitate surgical planning, improve student teaching, promote research and improve customer communication. The use of rapid prototyping is expected to increase as volumetric acquisition in MRI and ultrasound becomes more routine.

## 2. Materials and Methods

### 2.1. Animals

For this study, we utilized cadavers of 6 crossbreed cats (*Felis silvestris catus*, L.)—2 males and 4 females—each 2–3 years old and weighing about 3–3.5 kg, from the Zoonoses Service and Pest Control of Murcia, that were humanely euthanized for causes unrelated to this study. Specimens were then used for a contrast medium tomographic study of the liver. This project was supervised and approved by the ethics committee of the University of Murcia, Spain (REGA ES300305440012 CEEA:305/2017).

### 2.2. Computed Tomography Technique

Three feline cadavers were transported to the dissection room (Anatomical Laboratory, Department of Anatomy and Embryology, University of Murcia, Murcia, Spain) and pumping out of the vasculature was performed with 2% saline solution via the common carotid artery, which was then injected with a colored vulcanized latex (blue, red and green) (NV001, Ballons CP, Espinardo, Murcia, Spain) and with 50% hydrated barium sulfate (12 g/100 mL of distilled water) to analyze the venous, arterial and biliary system via the aorta, gallbladder and portal vein. CT scans were then performed at the Veterinary Hospital of the University of Murcia, Spain (General Electric HiSpeed dual 2-detector CT, General Electric Healthcare, Madrid, Spain). Different parameters and sequences were used for the arterial, venous and biliary (Table 1) study.

The DICOM image files generated by the CT scanner were analyzed with the AMIRA 5.6 (Thermo Fisher Scientific, Waltham, MA, USA) and OsiriX MD 13.0.2 (Pixmeo, Bernex, Switzerland) programs; subsequently, followed by different postprocessing algorithms’ images of maximum intensity projection (MIP), superficial and volumetric reconstructions (volume rendering) were obtained. Finally, arterial, venous and biliary 3D prints of the feline liver were obtained using Grabcad Print 1.71.7.21930 program (Waltham, MA, USA) and Stratasys F170-FDM printer (Los Angeles, CA, USA). ABS plastic and soluble support material were used for models and support material was subsequently dissolved in an additive water bath at 70 °C. Finally, printed models were painted with different colors in order to identify the different anatomical structures.

### 2.3. Cast Preparation and Corrosion Techniques [23]

#### 2.3.1. Precasting Procedure

The other three feline cadavers were transported to the dissection room (Anatomical Laboratory, Department of Anatomy and Embryology, University of Murcia, Spain) where the vasculature was pumped out via the common carotid artery and external jugular vein.

#### 2.3.2. Casting Procedure

Colored epoxy was injected as follows: blue for the venous system via caudal vena cava in reverse direction towards the liver, red for the arterial system via the descending aorta in reverse direction towards the heart and green for the biliary system via the gallbladder in reverse direction towards the liver and bile duct (clamped) (E20 plus BIODUR^®^*,* Biodur Products, Biodur, Heidelberg, Germany). The livers were then removed from the abdominal cavity. Caustic soda (sodium hydroxide) was used for the organic digestion process.

#### 2.3.3. Post-Casting Procedure

The tissues were corroded and washed with water to clean hepatic casts. Finally, casts were photographed to compare with CT images (AMIRA and OsiriX) and 3D prints.

## 3. Results

**Figure 1:**
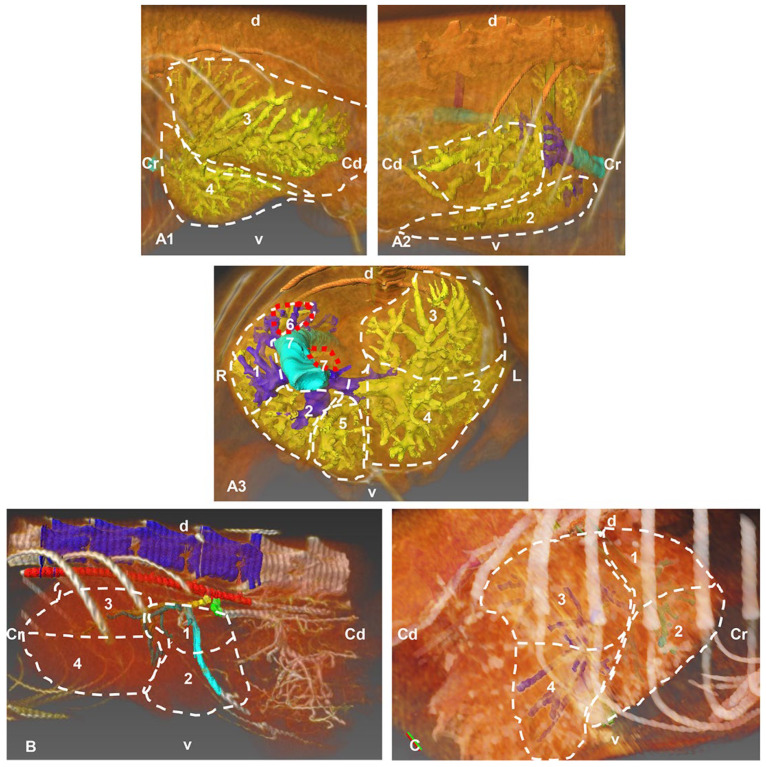
Images in which the automatic volumetric reconstruction of the liver lobes has been superimposed (highlighted with discontinuous lines) and with manual segmentation (venous, arterial and biliary reconstructions), carried out with the Amira program, showing the distribution by lobes of venous, arterial and biliary structures. (**A**) Portal venous system (yellow) of the hepatic veins (dark blue). (**A1**) Left side view. (**A2**) Right side view. (**A3**) Cranial view. (**B**) Hepatic arterial system (dark green), left side view. (**C**) Left biliary system (light blue) and right (dark blue), right side view. Cr: cranial; Cd: caudal; L: left; R: right; d: dorsal; v: ventral; 1: right lateral hepatic lobe; 2: right medial hepatic lobe; 3: left lateral hepatic lobe; 4: left medial hepatic lobe; 5: quadrate hepatic lobe; 6: caudate hepatic lobe—caudate process; 7: caudate hepatic lobe—papillary process.

Models of the liver parenchyma, its arteries, its veins and the network of bile ducts were developed by joining volumetric reconstruction by automatic volumetric segmentation and reconstruction provided by the AMIRA program. The course of each of these structures within each liver lobe was followed by superimposing the structures of the venous, arterial and biliary systems (Figure 1). Each of the structures was positioned in relation to its respective liver.

When the 3D model was produced, we were able to carefully analyze the structures at all levels, observing the correspondence with the images obtained in volume rendering of Amira and OsiriX, the segmentation and the 3D printed model.

The *arterial supply* of the liver is received from the hepatic artery, the course of which was traced from the celiac artery. We observed the other two branches of the celiac artery: the left gastric artery, in a central position, and the splenic artery to the left. The hepatic artery in the liver porta is divided into a right branch and a left branch.

The right branch emits a branch to the right hepatic lobe and ends in four sub-branches dorsocaudally positioned. Finally, the right branch divides into three branches that are directed to the right medial hepatic lobe and the gallbladder.

**Figure 2:**
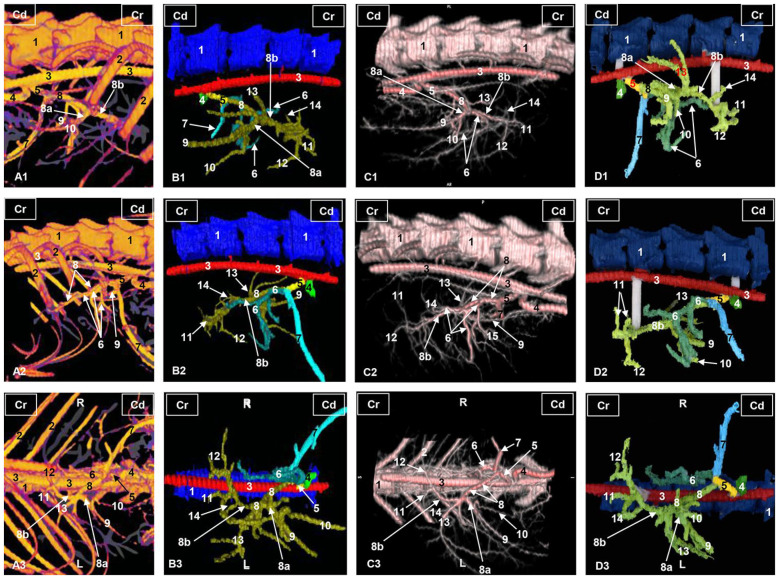
Three-dimensional reconstructions images of the liver arterial system in cats. (**A**) Amira volume rendering. (**B**) Amira segmentation reconstruction. (**C**) OsiriX volume rendering. (**D**) 3D printing. (**A1**–**D1**) Right side view. (**A2**–**D2**) Left side view. (**A3**–**D3**) Ventral view. R: right side; L: left side; Cr: cranial; Cd: caudal; 1: thoracic vertebrae; 2: ribs; 3: thoracic aorta—descending aorta; 4: cranial mesenteric artery; 5: celiac artery; 6: left gastric artery; 7: splenic artery; 8: hepatic artery; 8a: hepatic artery, right branch; 8b: hepatic artery, left branch; 9: hepatic artery, branch for the right lateral lobe; 10: hepatic artery, branch for the right medial lobe; 11: hepatic artery, branch for the left lateral hepatic lobe; 12: hepatic artery, branch for the left medial hepatic lobe and quadrate hepatic lobe; 13: hepatic artery, branch for the caudate hepatic lobe (caudate process); 14: hepatic artery, branch for the caudate hepatic lobe (papillary process).

The left branch sends a small dorsal branch towards the papillary process of the caudate lobe, two left medial branches continue and ventral branches travel to the left medial lobe and the quadrate lobe. The left branch finally divides into four branches which supply the left lateral lobe (Figure 2).

**Figure 3:**
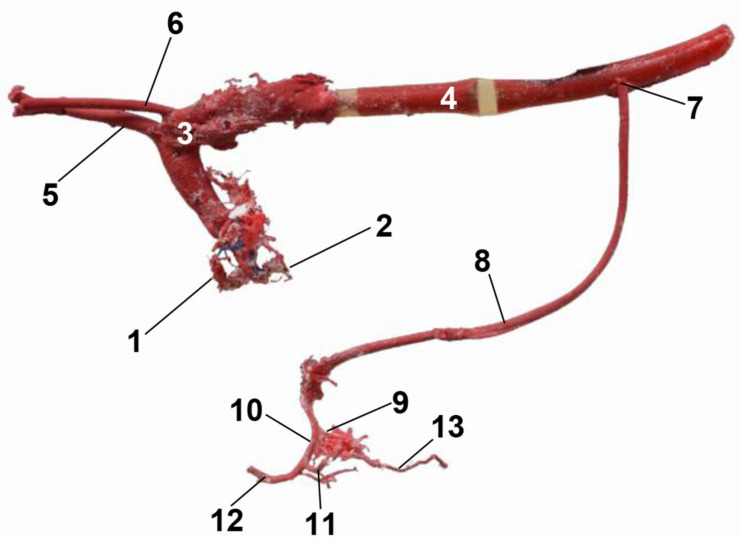
Cast of the aorta and its main branches. Celiac artery and hepatic artery to the liver. 1: right coronary artery; 2: left coronary artery; 3: aortic arch; 4: descending aorta—thoracic aorta; 5: brachiocephalic trunk; 6: left subclavian artery; 7: celiac artery; 8: hepatic artery; 9: right hepatic artery; 10: left hepatic artery; 11: left lateral hepatic artery; 12: left medial hepatic artery; 13: right lateral hepatic artery.

In the corrosion cast of the arterial supply, we can see only the division of the hepatic artery into the right and the left branches and its main branches (Figure 3).

**Figure 4:**
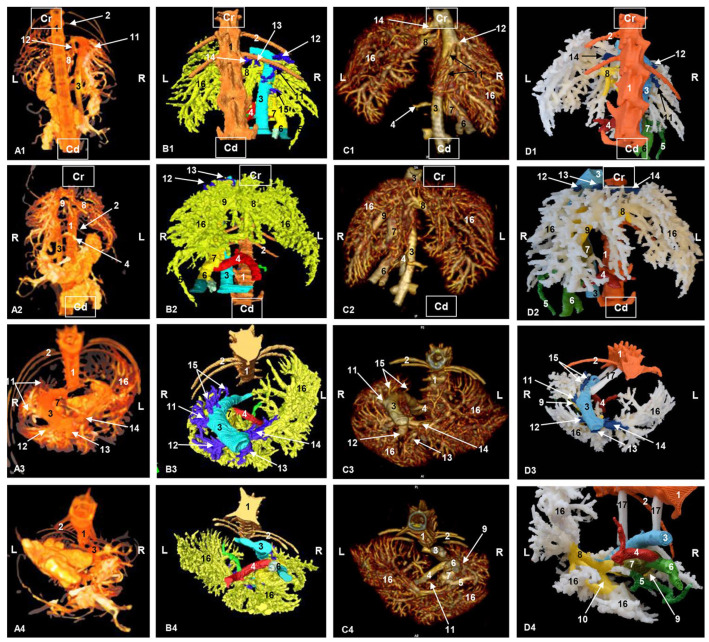
Three-dimensional reconstruction image of the venous system of the liver in cats. (**A**) Amira volume rendering. (**B**) Amira segmentation reconstruction. (**C**) OsiriX volume rendering. (**D**) 3D printing. (**A1**–**D1**) Dorsal view. (**A2**–**D2**) Ventral view. (**A3**–**D3**) Cranial view. (**A4**–**D4**) Caudal view. R: right side; L: left side; Cr: cranial; Cd: caudal. 1: thoracic vertebrae; 2: rib; 3: caudal vena cava; 4: splenic vein; 5: gastroduodenal vein; 6: cranial mesenteric vein; 7: portal vein; 8: left branch of portal vein; 9: right branch of the portal vein; 10: left branch of portal vein—transverse part; 11: right lateral hepatic vein; 12: right medial hepatic vein; 13: middle hepatic vein; 14: left hepatic vein; 15: accesory hepatic veins; 16: branching out of portal vein; 17: shank.

We distinguish two types of *venous drainage* in this study: functional and metabolic. The functional venous drainage conveys products of the digestive system through the portal vein to the liver. We first examined the main tributaries of the portal vein: gastroduodenal veins (right), cranial mesenteric vein (central, with respect to vena porta) (Figure 4) and splenic veins (left).

The portal vein splits into the left branch, which emits a first branch, and the transverse portion, which transports the blood to the quadrate lobe and the caudate lobe (papillary process). The left branch continues towards the right medial lobe and to the left medial lobe. A right branch that divides is directed towards the right lateral lobe and the caudate lobe (caudate process). From these branches, portal blood travels to all the liver lobes so that the nutrients are processed by the hepatocytes (Figure 4).

A corrosion cast of the portal vein could not be performed successfully due to the high density of epoxy material and the difficulty to clear the portal vein and its intrahepatic branches during precast procedure.

The principal *hepatic veins* are associated with metabolic venous drainage. The blood from the left lateral lobe is drained by the left hepatic vein, while the left medial lobe, quadrate lobe and caudate lobe (papillary process) are drained by the middle hepatic vein. The right medial lobe is drained by the right medial hepatic vein while the right lateral hepatic lobe and the caudate lobe (papillary process) are drained by the lateral right hepatic vein and the accessory hepatic veins; these latter vessels, being of small caliber, empty directly into the caudal vena cava. All this blood flows into the caudal vena cava (Figure 4).

**Figure 5:**
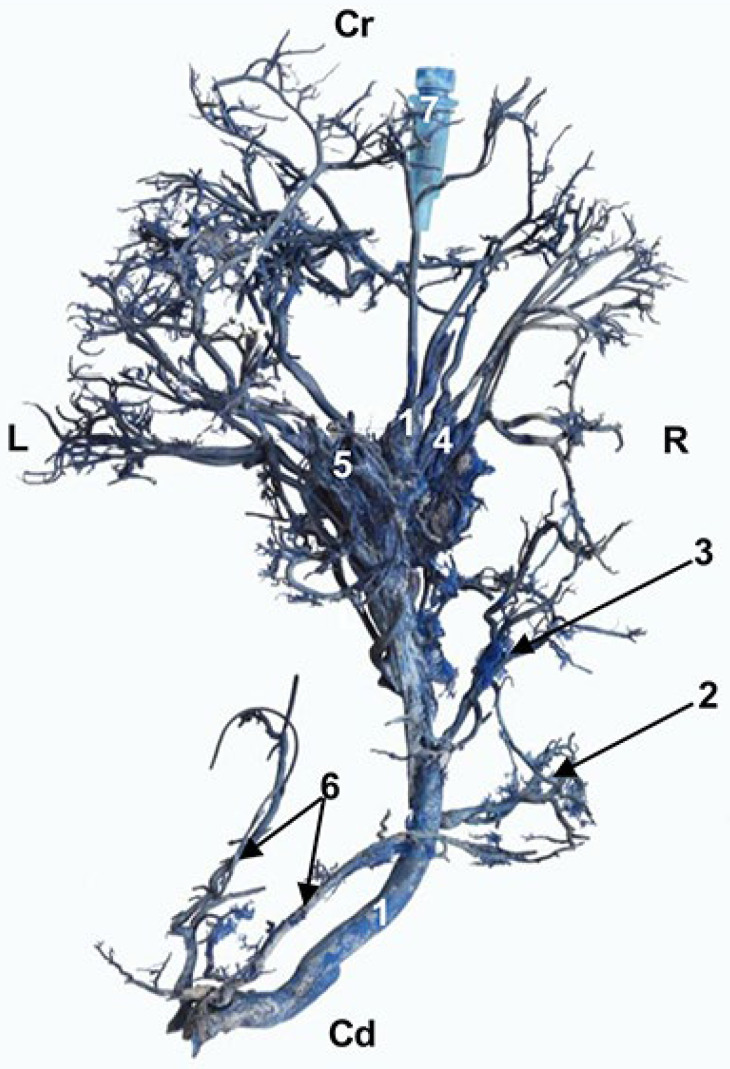
Cast of hepatic veins. The needle is still inserted inside the thoracic caudal vena cava. Dorsal view; Cr: cranial; Cd: caudal; L: left; R: right; 1: caudal vena cava; 2: right lateral hepatic vein; 3: right medial hepatic vein; 4: middle hepatic vein; 5: left hepatic vein; 6: renal veins (right and left); 7: needle.

In the corrosion cast, we were able to observe the course of the liver veins from the lobes to their connections to the caudal vena cava (Figure 5).

**Figure 6:**
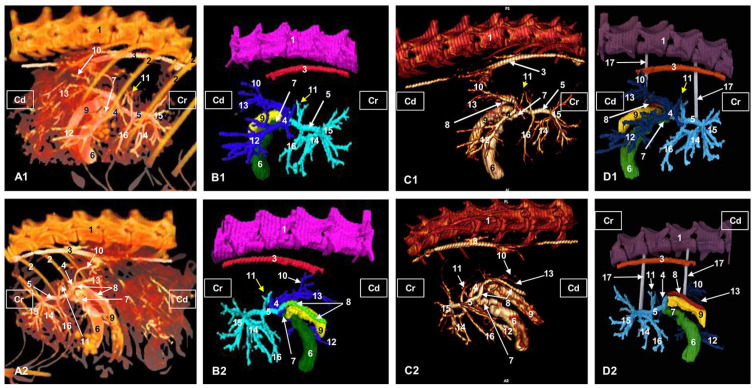
Three-dimensional reconstruction images of the biliary system in cats. (**A**) Amira volume rendering. (**B**) Amira segmentation reconstruction. (**C**) OsiriX volume rendering. (**D**) 3D printing. (**A1**–**D1**) Right side view. (**A2**–**D2**) Left side view. Cr: cranial; Cd: caudal; 1: thoracic vertebrae; 2: ribs; 3: thoracic aorta—descending aorta; 4: right hepatic duct (dark blue); 5: left hepatic duct (clear blue); 6: gallbladder; 7: cystic duct: 8: bile (choledochal) duct; 9: duodenum; 10: intrahepatic bile ducts from the caudate hepatic lobe, caudate process; 11: intrahepatic bile ducts from the caudate hepatic lobe, papillary process; 12: intrahepatic bile ducts from the right medial hepatic lobe; 13: intrahepatic bile ducts from the right lateral hepatic lobe; 14: intrahepatic bile ducts from the left medial hepatic lobe; 15: intrahepatic bile ducts from the left lateral hepatic lobe;16: intrahepatic bile ducts from the quadrate hepatic lobe; 17: shank.

**Figure 7:**
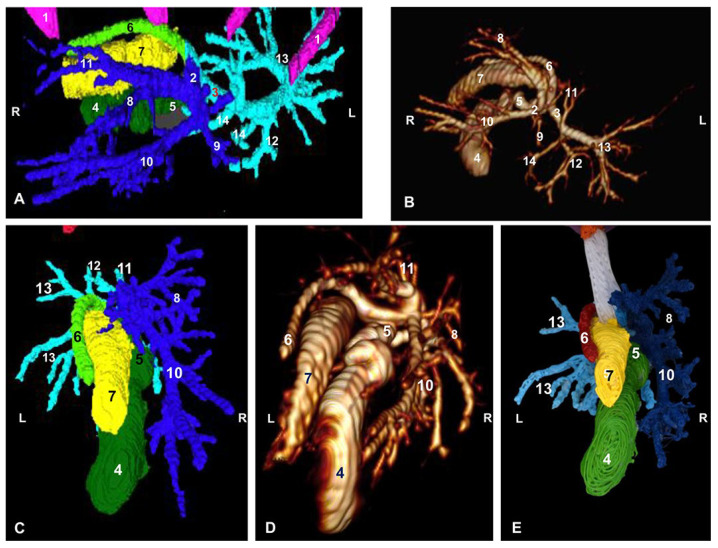
Three-dimensional reconstruction images of the biliary system in cats. (**A**,**C**) Amira segmentation reconstruction. (**B**,**D**) OsiriX volume rendering. (**E**) 3D printing. (**A**,**B**) Dorsal view. (**C**–**E**) Caudal view. R: right side; L: left side; 1: ribs; 2: right hepatic duct (dark blue); 3: left hepatic duct (clear blue); 4: gallbladder; 5: cystic duct; 6: bile (choledochal) duct; 7: descending duodenum; 8: intrahepatic bile ducts from the caudate lobe, caudate process; 9: intrahepatic bile ducts from caudate lobe, papillary process; 10: intrahepatic bile ducts from the right medial hepatic lobe; 11: intrahepatic bile ducts from the right lateral hepatic lobe; 12: intrahepatic bile ducts from the left medial hepatic lobe; 13: intrahepatic bile ducts from the left lateral hepatic lobe; 14: intrahepatic bile ducts from the quadrate hepatic lobe.

Bile formed from the hepatocytes flows through the intrahepatic *bile ducts* from the small peripheral ramifications of the casts (canaliculi). Intrahepatic ducts from the right lateral and right medial hepatic lobes and the caudate lobe (caudate and papillary processes) (Figure 6 and Figure 7) drain the intrahepatic right liver conduit (dark blue) (Figure 6). The bile from the left lateral, left medial and quadrate hepatic lobes, along with a branch from the caudate lobe (caudate process) (Figure 6 and Figure 7) drain the intrahepatic left liver conduit (clear blue) (Figure 6). Intrahepatic ducts emerge from the liver as extrahepatic ducts. The extrahepatic left liver duct (clear blue) connects with the cystic duct. The extrahepatic right liver duct (dark blue) connects with the cystic duct; both the extrahepatic left cystic duct and the hepatic duct constitute the bile duct (ductus choledochus) (Figure 6). The bile duct joins the descending duodenum at the major duodenal papilla (Figure 6 and Figure 7).

**Figure 8:**
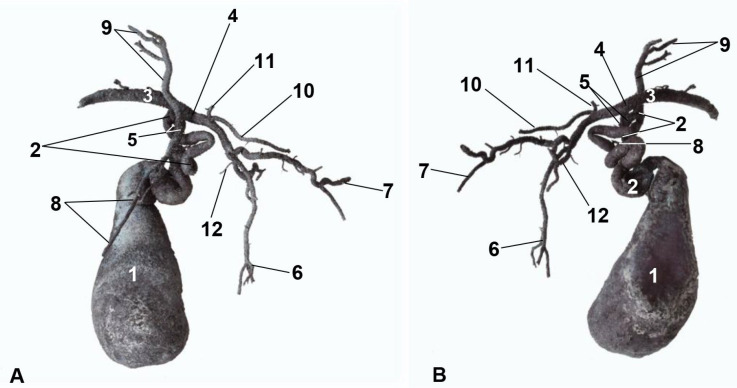
(**A**) Right side view. (**B**) Left side view. 1: gallbladder; 2: cystic duct; 3: bile (choledochal) duct; 4: left hepatic duct; 5: right hepatic duct; 6: intrahepatic bile ducts of the left lateral hepatic lobe; 7: intrahepatic bile ducts of the left medial hepatic lobe; 8: intrahepatic bile ducts of the right lateral hepatic lobe; 9: intrahepatic bile ducts of the right medial hepatic lobe; 10: intrahepatic bile ducts of the quadrate hepatic lobe; 11: intrahepatic bile ducts of the caudate hepatic lobe, caudate process; 12: intrahepatic bile ducts of the caudate hepatic lobe, papillary process.

The corrosion cast of the bile system allowed us to observe the same disposition as in three-dimensional reconstructions (Figure 8).

## 4. Discussion

Studies related to feline abdominal anatomy are still scarce, despite the increase in feline patients in veterinary clinics. This study of feline liver parenchyma is structured in three parts: arterial, venous and biliary.

The arterial circuit generally follows the pattern described in carnivores by Sandoval; Nickel et al. [24,25], who described the hepatic artery, before entering the liver in carnivores, as dividing into lateral and medial right and left branches. The cystic artery arises from the medial left branch in carnivores. In our study of cats, we did not identify the origin of the cystic artery. Mari and Acocella; Schaller [26,27] used schematic drawings to describe the blood supply to a carnivore’s liver. Their study indicated that the right hepatic artery arises from a right lateral branch towards the right lobe. From the right lobe, a branch travels towards the caudate lobe (caudate process).

However, in cats, the artery supplying the caudate process leaves the right hepatic artery before giving off lateral and medial branches. According to Mari and Acocella [26] and Schaller [27], in carnivores, the right medial branch leaves the hepatic artery before its bifurcation, which agrees with our current study.

We distinguished a functional portal venous circuit (afferent) to the liver that supplies nutrients as described in Nickel et al. [25,28] and another hepatic circuit (efferent), responsible for the nutrition of liver cells, provided by the hepatic artery. Metabolites travel towards the hepatic veins from the centrilobular vein to the caudal vena cava together with the processed nutrients coming from the portal vein.

Our study found that the gastroduodenal, cranial mesenteric and splenic veins combined to form the hepatic portal vein, which is in agreement with the results of Sandoval [24] and Nickel et al. [25]. Second, Sandoval; Nickel et al.; Mari and Acocella; Schaller; Nickel et al. [24,25,26,27,28] divided the trunk of the portal vein at the hepatic porta into two branches (right and left); the less developed right towards the right lobe and caudate lobe, and the thicker left towards the left lobe and quadrate lobe, a general pattern for all domestic species.

According to Schaller [27] and Nickel et al. [28], the division of the canine portal vein produces a right branch of the portal vein for the right lateral lobe and the caudate lobe (caudate process), which concurs with our findings. However, we did not identify, in cats, the transverse portion or the umbilical portion referred to Schaller [27] and Nickel et al. [28] in carnivores, pigs, horses and ruminants. Other studies carried out by Gaschen; Nickel et al.; Scavelli et al. [1,28,29] and Rojo et al. [22] did not follow the branches of the portal vein within the liver. Metzger et al. [6] analyzed the portal venous system in cats. Our study agrees with one of the six schematic representations of the intrahepatic portal venous system which divided the portal vein in two main branches at the level of the porta.

With respect to the efferent liver circuit, we agree with the views expressed by De Sordi et al. [23]. The hepatic veins (right (lateral and medial), middle and left) and hepatic accessory veins observed in dogs were also observed in the cats in our study, although Metzger et al.; Schaller; Nickel et al. [6,27,28] do not describe any division on the right. However, they observed and described the middle and left veins, but not the accessory liver veins that travel independently to the vena cava, which were observed in our study of cats (corrosion and 3D casts).

Finally, Ursic et al. [30] carried out a study of anatomical changes in the portal vein supply in dogs and commented on the complexity of the drainage system of the portal vein in cats.

As for the biliary system, we agree with the description by Nickel et al. [28] of the circuit that transfers bile from the liver lobes to the gallbladder and bile duct. By using contrast CT to obtain volumetric reconstructions and three-dimensional impressions, our study described the circuit from the lobes to the confluence of the right and left hepatic ducts in cats. Our findings indicate that the bile produced in the right hepatic and caudate lobes enters the right hepatic duct. Bile from the papillary process goes to both the right and the left hepatic ducts [31]. The bile produced in the left hepatic lobe (lateral and medial) and quadrate lobe enters the left hepatic duct [31].

Other works, such as Sandoval; [24] and Schaller [27], provide only schematic drawings. CT research on dogs, such as Teixeira et al. [32], does not analyze the biliary circuit. We do not concur that the biliary pattern described in carnivores by Sandoval [24] applies to cats.

In addition, in our study, the right hepatic duct flows into the cystic duct; the left and cystic duct form the bile duct. These findings do not coincide with Sandoval [24], but they are in agreement with Crouch; Hudson and Hamilton [33,34] and with some of the 30 cats studied in Bragulla and Vollmerhaus [31].

Likewise, we agree with Sandoval; Schaller; Bragulla and Vollmerhaus; Hudson and Hamilton [24,27,31,34] that the common hepatic duct is absent and that the common bile duct is present in cats. However, Hudson and Hamilton [34] refers to the common bile duct as the common hepatic duct.

In Bragulla and Vollmerhaus [31], the authors observed that all the bile collected by the right and left hepatic ducts empties into the cystic duct. We agree that the right hepatic duct almost always empties into the cystic duct and, finally, that the cystic and left hepatic duct form the common bile duct. In the two cats in our study, this pattern was shown in the extrahepatic biliary system. However, Bragulla and Vollmerhaus [31] describe 17 variations of this system in the 30 cases studied through corrosion, which makes it difficult to establish a pattern in cats. Even so, we consider that these are important data to consider for future studies of the feline biliary system.

## 5. Conclusions

The combination of the CTA and the anatomical images injected with latex and epoxy by the caudal vena cava, hepatic artery and gallbladder holder allowed us to differentiate arterial irrigation, venous drainage and bile circulation in the feline liver.

The correlation between the images of OsiriX, Amira and the 3D impression are a good example of what a normal three-dimensional model of the vascularization of the feline liver can contribute as help to veterinary clinical work. In future, three -dimensional printed models of portosystemic referrals can be created to help doctors understand these anomalies for subsequent surgical resolution and could contribute to the teaching of normal anatomy.

The 3D imaging and printing of the hepatic vascular and biliary systems will be a useful resource for the clinician for the preoperative planning of different surgeries (shunts, liver masses, etc.) in feline patients.

## Figures and Tables

**Table 1:** Parameters used in arterial, venous and biliary system study.

Study Type	DT	ST	SR	Pitch	Kv	mA	SP	SS	STh	RAlg
**Arterial**	M2DA	4′21′’	0.42	1	120	120	Transverse	Craniocaudal	0.6 mm	Soft Tissue
**Venous**	M2DA	13’27”	0.42	1	120	80	Transverse	Craniocaudal	0.6 mm	Soft Tissue
**Biliary**	M2DA	8’13’’	0.42	1	120	120	Transverse	Craniocaudal	0.6 mm	Soft Tissue

DT: detector type; ST: scan time; SR: spatial resolution; Kv: kilo voltage; mA: mile amperage; SP: sectional plane; SS: scan sequence; STh: slice thickness; RAlg.: reconstruction algorithm; M2DA: multislice 2 detector array.

## Data Availability

Not applicable.
